# Profiles of domestic violence victims and perpetrators

**DOI:** 10.1192/j.eurpsy.2026.11290

**Published:** 2026-07-31

**Authors:** S. Omri, N. Bouattour, H. Dhouib, N. Charfi, I. Gassara, R. Feki, M. Mâalej, M. Mâalej, W. Ben Amar, N. Smaoui, L. Zouari

**Affiliations:** 1 Psychiatry C, Hedi Chaker University Hospital; 2 Forensic Medecine, Habib Bourguiba University Hospital, Sfax, Tunisia

## Abstract

**Introduction:**

Domestic violence (DV) is increasingly high in our society. A Tunisian survey conducted by the National Family Planning Office in 2010 pinpointed the frequency and the recurrence of this phenomenon.

**Objectives:**

To describe the demographics of the women victims and their spouses or ex-spouses.

**Methods:**

A descriptive cross-sectional study was conducted over a period of three months on women victims of domestic violence examined at the forensic medicine department of the Habib Bourguiba University Hospital in Sfax. All data was collected using self-reporting questionnaires, which were filled up by those women themselves. Profiles of the women sample (and the perpetrators) were analysed via the PASW Statistics 18 Advanced Statistical procedure.

**Results:**

We included 60 female victims of DV in the study. The median age of victims was 33.5 years (IQR [25.75]) with extremes ranging from 18 to 65 years. The median age when they got wed was 23 years old (IQR [25,75]). Only 5% of the victims were illiterate. The majority, 42%, went to secondary school, followed by academic education,28%, and primary school 25%. Urban geographic origin was slightly higher among the victims, with 52%. As for occupation, 53.3% were housewives, 30% were laborers, 13.3% were civil servants or senior executives, and 3.3% were students. Regarding personal habits, 91.7% had no specific habit.

The median age of the abusive partner was 38.5 years (IQR [25–75]). Most of them were manual laborers (46.7%), 4% had a criminal record, and 15% had a regular consumption of weed.

According to the victims, the choice of their spouses was based on love in 62% of the cases. The average engagement period lasted 12 months (range: 12–22.5 months). The mean age difference between spouses was 4.5 years (3-7 years). In three cases, the woman was older than her spouse. The socioeconomic status of the couple was low in 13 cases (21.7%). Seven years was the mean duration of the wedding at the time of the current assault, with extremes ranging from 4 months to 40 years. The most reported motive for the aggression was financial difficulties (30%).

**Conclusions:**

The study highlights a diverse demographic profile of women victims of domestic violence, predominantly young, educated, and mostly housewives. Their abusive partner was generally older, mostly a manual laborers, with a minority having a criminal record or substance use. Financial difficulties emerged as the leading cause of aggression, emphasizing the complex socio-economic factors involved in these cases.

**Disclosure of Interest:**

None Declared

